# Oxidized Glutathione Increases Delta-Subunit Expressing Epithelial Sodium Channel Activity in *Xenopus laevis* Oocytes

**Published:** 2020-05-25

**Authors:** Garett J. Grant, Camila Coca, Xing-Ming Zhao, My N. Helms

**Affiliations:** 1 Department of Internal Medicine, Pulmonary Division, University of Utah, Salt Lake City, UT, USA; 2 Institute of Science and Technology for Brain Inspired Intelligence, Fudan University, Shanghai, China

**Keywords:** Two Electrode Voltage Clamp (TEV), oocytes, S-glutathionylation, glutathione disulfide, Cys thiols

## Abstract

Epithelial sodium channels (ENaC) are heterotrimeric structures, made up of α, β, and γ subunits, and play an important role in maintaining fluid homeostasis. When δ-ENaC subunits are expressed in place of (or in addition to) the α-ENaC subunit alongside β- and γ- subunits, fundamental changes in the biophysical properties of ENaC can be observed. Using human ENaC cRNA constructs and the *Xenopas laevis* oocyte expression system, we show that oxidized glutathione (GSSG) differently effects αβγ-ENaC and αβγ-ENaC current. GSSG (400 μM) significantly decreased normalized whole cell current in oocytes expressing αβγ-ENaC, and conversely increased whole cell current in δ1βγ-ENaC and δ2βγ-ENaC expressing oocytes. GSSG treatment increased current in oocytes expressing all four subunits. Western blot and PCR analysis show that human small airway epithelial cells (hSAEC) express canonical αβγ-subunits alongside δ-ENaC subunits. Differences in single channel responses to GSSG in hSAECs indicate that airway epithelia redox sensitivity may depend on whether δ- or α- subunits assemble in the membrane. *In silico* analysis predict that six Cys amino acids in the δ-ENaC extracellular loop, and a single Cys in the N-terminal domain, are susceptible to post-translational modification by GSSG. Additional studies are needed to better understand the molecular regulation and pathophysiological roles of oxidized glutathione and δ-ENaC in lung disorders.

## Introduction

Amiloride-sensitive epithelial sodium channels (ENaC) are heterotrimeric ion channels that play important roles in maintaining fluid and cell homeostasis [1,2]. In 1993, α, β, and γ were the first ENaC subunits cloned and are therefore considered the canonical subunits comprising sodium channels widely expressed in the distal nephron, secretory glands, colon, and airways of vertebrates and invertebrates [3–5]. More recently, a primate specific δ-ENaC subunit has been identified in brain, pancreas, testis, ovary [6], and lung tissue [7]. Two splice variants of δ-ENaC (termed δ1 and δ2) have been described in alveolar epithelial cells [7]. Both isoforms can be expressed in the same alveolar cells, albeit δ2 is expressed at lower levels [7].

Macroscopic current is often measured to study the biophysical and pharmacological features of heteromultimeric channels. For example, heterologous expression of ENaC cRNA in Xenopus oocytes has shown that αβγ channels generate greater whole cell current *vs*. αβγ channels [8]. This difference between δ and α comprising ENaC channels may be attributed, in part, to the δ subunit’s lack of sodium self-inhibition (which is well characterized in αβγ channels) [9–11]. Pharmacological studies reveal several additional divergent responses between αβγ and αβγ macroscopic current following exposure to the same experimental treatments [12]. The specific aim of this study was to better understand GSSG regulation of αβγ and δβγ channels and determine whether ENaC subunit composition influences net Na^+^ transport across an epithelium under pro-oxidizing conditions.

Oxidative stress is defined as the imbalance between oxidant and antioxidant levels that lead to disruption of redox signalling and control [13]. In the lungs, reduced glutathione (GSH) is abundantly available and serves as a reducing equivalent to neutralize reactive oxygen species. The utilization of GSH leads to a decrease in its bioavailability and accordingly, an increase in its oxidized form (GSSG; also termed glutathione disulfide). As such, the ratio of lung GSH-to-GSSG levels can serve as a biomarker for oxidative stress and lung injury [14]. Under pro-oxidizing conditions, elevated levels of GSSG participates in the reversible post-translational modification of Cysteine thiols, which may mitigate or mediate oxidative damage depending on the target protein [15]. In a previous study, we showed that tracheal instillation of GSSG into mouse lung significantly decreased the rate of alveolar fluid clearance and attenuated ENaC activity in mouse alveolar type 1 and type 2 cells [16]. Although these studies indicate that GSSG inhibits canonical ENaC activity, whether GSSG would alter solute transport in the human airway epithelium is unclear. Given the importance of regulating Na^+^ transport in maintaining lung fluid homeostasis, and the ability of oxidative stress to alter GSH-to-GSSG ratios in the lung, it is important to better understand the role that GSSG plays in regulating ENaC activity under pro-oxidizing lung disease.

Given the lack of δ-ENaC subunit expression in mouse lung, we modelled and evaluated the effect of GSSG on δ1βγ and δ2βγ channels that were heterologously expressed in oocytes, and in primary human small airway epithelial cells herein. Systematic evaluation of ENaC subunit composition and its impact on net fluid balance is important for understanding whether ENaC is a viable therapeutic target for lung injury. Our study showed that glutathione disulfide effected αβγ and δβγ-ENaC whole cell current in an opposing manner. The study also identified the δ-ENaC Cys residues that may be post-translationally modified by GSSG were identified using *in silico* analysis. Together, these studies serve to increase our scientific understanding of the molecular underpinnings of lung disease caused by ion channel dysfunction and oxidative stress.

## Materials and Methods

### Reagents and chemicals

Unless otherwise stated, all chemicals and reagents were purchased from Sigma-Aldrich, St. Louis, MO. The extracellular KCM-211 solution used for voltage-clamp experiments contained the following (in mM): 98 NaCl, 2 KCl, 1 CaCl_2_, 1 MgCl_2_, 5 HEPES; pH adjusted to 7.6 with NaOH. Glutathione disulfide (GSSG) and glutathione (GSH) were solubilized in KCM-211 solution.

### Vectors

Human α, β, γ, δ1 and δ2 ENaC cDNAs cloned in pTNT Vector (Promega, Madison, WI) were kind gifts from Dr. Mike Althaus (Bonn-Rhein-Sieg University of Applied Sciences, Germany). From this vector, cRNA encoding ENaC subunits were prepared by linearizing the plasmid with BamH1 followed by in vitro transcription using the mMessage mMachine SP6 kit (Ambion, Austin, TX).

### Xenopus oocytes

Defolliculated stage V Xenopus oocytes were purchased from Ecocyte Bioscience (Austin, TX). Oocytes were injected with 5–30 ng of ENaC cRNA in a final volume of 17 nL of solution containing a 1:1:1 ratio of either α, β, γ-ENaC or δ, β, γ-ENaC. After cRNA injection, oocytes were maintained in Super Barth’s saline solution at 16°C. Super Barth’s saline solution is composed of (in mM): 88 NaCl, 5 KCl, 2.4 NaHCO_3_,10 HEPES, 0.33 Ca(NO_3_)_2_, 0.41 CaCl_2_, 1 MgSO_4_, 100 pyruvate and supplemented with: 100 μg/mL Amikacin, 25 μg/mL Ciprofloxacin, 50 μg/mL gentamycin; pH adjusted to 7.4 with NaOH. All experiments were performed within 24–48 hrs post cRNA injection at room temperature.

### Two electrode voltage clamp recordings

Whole cell ENaC currents were recorded using agarose-cushion microelectrodes and standard two-electrode voltage-clamp techniques [17]. Oocytes were placed in a Plexiglas chamber and were continuously perfused with extracellular KCM-211 solution at a flow rate of 75 mL/hour. Recording electrode resistances were between 0.5–1.o MΩ. Test potentials that ranged between −80 mV to +60 mV applied in 10mV increments were applied to injected oocytes. GeneClamp 500 amplifier, Digidata 1322A, and pCLAMP ver. 8.2 software (Molecular Devices, San Jose, CA) were used for data acquisition.

### Tissue Culture

Cryopreserved human small airway epithelial cells (SAEC) were purchased from Lonza Bioscience (Alpharetta, GA) and cultured in a 37°C humidified 5% CO_2_ incubator. The cells were cultured per supplier protocol using Lonza SAGM Small Airway Epithelial Cell Growth Medium and supplements. All experiments were performed between SAEC passages 1–4. For electrophysiological studies, cells were cultured on Costar Snapwell (Kennebtmk, ME) clear permeable supports with 0.4 μM polyester membrane to confluency.

### Single Channel Patch Clamp Recordings

ENaC activity was recorded using standard single channel patch clamp analysis in the cell-attatched configuration. Micropipettes were pulled with a two-stage Narishige PC-10 vertical puller (Narishige International, Amityville, NY) from filamented borosilicate glass capillaries purchased from World Precision Instruments (Sarasota, FL). Micropipette resistances were between 5–9 MΩ when filled and immersed in patch solution composed of (in mM): 140 NaCl, 5 KCl,1 CaCl_2_, 1 MgCl_2_, and 10 HEPES, adjusted to pH 7.40 with NaOH. Channel currents were sampled at 1 kHz with an Axopatch 1B patch amplifier (Molecular Devices) and filtered at 200 Hz with a low-pass Bessel filter. Continuous single channel activity was recorded and then analyzed using Pclamp 10 software for channel open probability (Po) and number (N) of active channels in SAEC apical membrane. Chord conductances (γ), were calculated from hyperpolarized and depolarized potentials.

### Immunohistochemistry

SAECs were seeded at equal density on poly-D-Lysine coated 12 mm round glass coverslips (Discovery Labware, Bedford, MA) for immunohistochemistry. Cells were fixed using periodate-lysine-paraformaldehyde for 10 min and then permeabilized with 0.5% Nonidet P-40 for 15 min, followed by incubation in 0.5% BSA and 0.1% gelatin in PBS. Primary α, β, and γ- ENaC antibodies RRID: AB_10640131; AB_10644173; AB_10640369, respectively, were purchased from StressMarq (Biosciences, Victoria, British Columbia). Primary δ-ENaC antibody (catalog item: ab196737) was purchased from Abeam (Cambridge MA). Antibodies were diluted 1:350 in PBS + 1X azide + 1% BSA and then applied to SAECs at room temperature (RT) for 1 hr, followed by additional labelling by the secondary goat anti-rabbit IgG conjugated to Alexa Fluor 488 (RRID:AB_2630356, purchased from Abcam) diluted 1:250 in PBS + 1X azide + 1% BSA 1 hr, RT). Nuclear DNA was labelled with mounting medium containing 4',6-diamidino-2-phenylindole (DAPI), purchased from Abcam. Confocal imaging was performed at The University of Utah Health Science Center Cell Imaging Core using a Nikon A1R confocal laser microscope system and NIH Image J software with Fiji plug-ins.

### Polymerase Chain Reaction

Total RNA was extracted from SAECs using a RNeasy isolation kit (Qiagen, Germantown, USA) following the protocol of the manufacturer. RNA was then treated with DNAse I and reverse-transcribed using Superscript II RNaseH-reverse transcriptase (Invitrogen, Carlsbad, CA). The level of δ ENaC subunit mRNA expression was determined using the following forward and reverse primers: GGT GCC AGT GAC GCT CAA AGA (forward) CGA AGC ATG GAC GGG AGA ATG (reverse). The α, β, and γ ENaC primers were previously reported in [18]. Conventional and nested PCR products were sequenced to verify subunit identify (DNA Sequencing Core Facility, University of Utah; sequencing data not shown). Quantitative real-time PCR was performed using a QuantStudio3 system and analyzed using associated software (ThermoFisher, Waltham, MA).

### *In silico* prediction of S-glutathionylation sites

S-glutathionylation is the reversible formation of mixed disulfides between GSSG and Cys thiols in proteins. Whether a Cys thiol is likely to be post-translationally modified in this manner can be predicted with a high degree of certainty using machine learning methods based on protein sequence data [16,19]. Briefly, *in silico* results were obtained with an AUC (area under ROC curve) score of 0.879 in 5-fold cross-validation [19]. We employed this bioinformatics method for predicting S-glutathionylation based on δ1 and δ2 ENaC protein sequence data obtained from The Universal Protein Resource database (www.uniprot.org entry P51172.1 and P51172.2, respectively).

### Data Analysis and Statistical Evaluation

pCLAMP 10.0 (Molecular Devices) and ORIGIN ver. 8.6 software (OriginLab, Northampton, MA) were used for offline analysis of digitized data. The amplitude of currents measured at −60 mV or −80 mV were normalized to its maximum value at the −20 mV test pulse. In this way, we are able to make whole cell comparisons amongst different experimental groups and batches of cells. Raw and normalized current reported; summary of normalized data reported as mean ± standard error (SE). Single comparisons were performed using paired Studenťs *t* tests in oocytes before and after treatment. Multiple comparisons were performed using one-way analysis of variance followed by the Bonferroni post-hoc test for pair-wise comparisons; p values of ≤ 0.05 were considered statistically significant.

## Results

The biophysical and pharmacological features of sodium channels comprised of αβγ and δβγ-ENaC subunits can be distinguished using a heterologous expression system coupled with electrophysiological recordings. Using this approach, we showed that αβγ-ENaC containing channels respond to GSSG treatment with changes in whole cell current that are distinct from δβγ-ENaC expressing channels. Furthermore, single channel analysis of GSSG treated hSAECs revealed discrete increase and decrease changes in ENaC open probability that would not be easily detected using any other approach. *In silico* analysis of α- and δ- sequences offers insight into ENaC domains expressing conserved Cys residues that may be regulated by GSSG under oxidative stress to control net solute transport.

### GSSG decreases αβγ-ENaC

**Figure 1.**
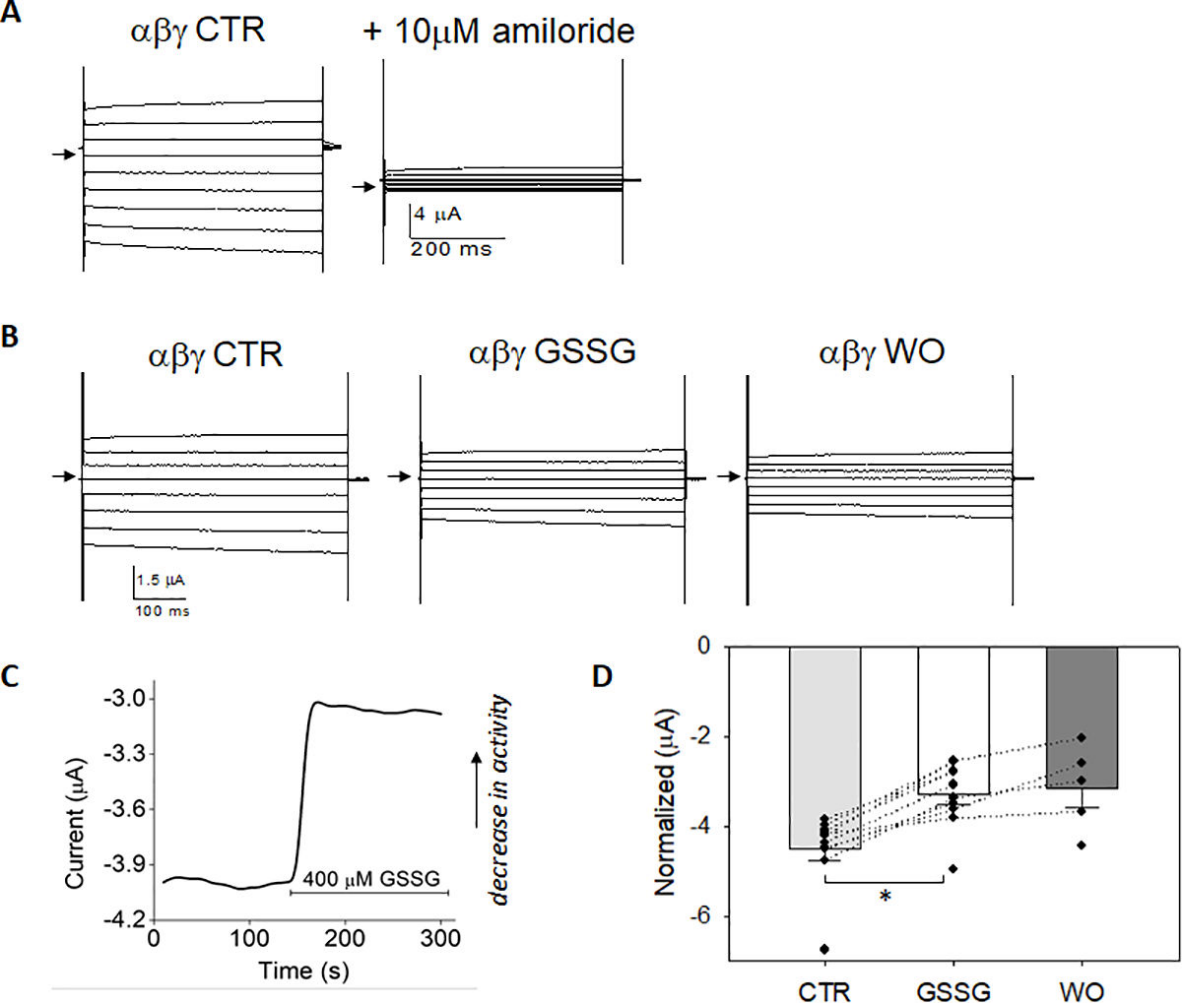
Glutathione disulfide (400 μM) decreases αβγ-ENaC whole cell current **A)** Amiloride-sensitive whole cell current recording from oocytes expressing WT human αβγ-ENaC; arrow indicates 0 current. Representative traces shown from test pulses between 100 mV to +60 mV in 20 mV increments. **B)** 400 μM GSSG significantly decreases αβγ-ENaC whole cell current (middle panel) without a change in current following wash out (WO) in the same cell recording. **C)** Representative time course and whole cell trace of αβγ- ENaC current at −60 mV before and during GSSG treatment where indicated. **D)** Normalized CTR and GSSG treated αβγ-ENaC whole cell current showing GSSG inhibition of activity from −4.5 ± 0.26 μA to −3.3 ± 0.23 μA; n=10 independent observations from 3 different oocyte preparations, *=*p*<0.01 using Paired *p*-test comparisons in each cell before and after GSSG perfusion.

Figure 1A shows amiloride-sensitive whole cell current measured in oocytes expressing human αβγ-ENaC cRNA in a 1:1:1 ratio at test potentials between −100mV through +60 mV and shown in 20mV step increments. Figures 1B, 1C are representative voltage-clamp studies obtained from αβγ-ENaC injected oocyte before and after GSSG (400 μM) perfusion, and following GSSG washout (WO) conducted sequentially in the same cell. GSSG decreased whole cell current at all clamped potentials (1B, middle panel); the washout period (1B, right panel) did not show further decline in whole cell current during washout. Figure 1C is a representative trace showing that GSSG significantly decreased αβγ-ENaC whole cell current within 10 seconds, which is equivalent to one sweep of the voltage clamp protocol. Conventionally, downward deflections represent inward transport of cations. In summary, Figure 1D shows normalized αβγ-ENaC whole cell current under control (5 min), GSSG [400 μM GSSG; 5 min], and washout (5 min) recordings; GSSG decreased normalized current from −4.5 ± 0.26 μAto −3.3 ± 0.23 μA, with no further run-down of channel activity during the washout, in 10 independent recordings from 3 different batches of oocytes prepared.

### αβγ-ENaC current is activated by GSSG in oocyte whole cell recordings

**Figure 2.**
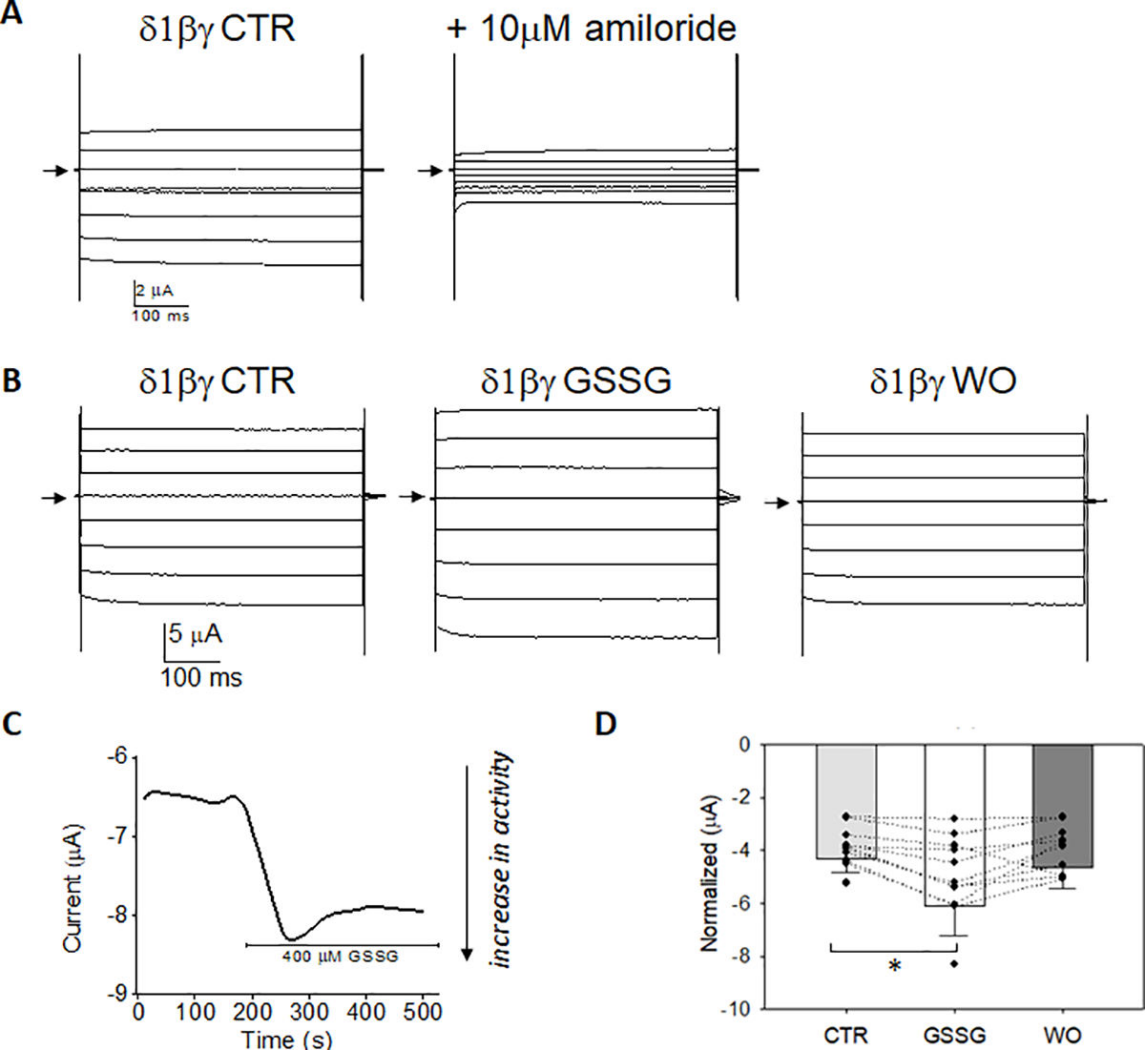
Glutathione disulfide (400 μM) increases δ1βγ-ENaC whole cell current **A)** Representative amiloride-sensitive δ1βγ-ENaC whole cell current; arrow indicates 0 current. Test pulses between −80 mV to +40 mV shown in 20 mV increments. **B)** GSSG perfusion increases δ1βγ-ENaC whole cell current and returns to basal levels following wash out (WO) in the same cell. **C)** Representative time course trace of GSSG activation of δ1βγ-ENaC; −60 mV holding potential. **D)** Normalized current of CTR, GSSG, and WO treated δ1βγ-ENaC expressing oocytes at −80 mV, wherein δ1βγ-ENaC current increased from −4.3 ± 0.50 μA to −6.0 ± 1.16 μA; n=12 independent observations from 3 different oocyte preparations, *=*p*<0.05 using Paired *t*-test comparisons.

**Figure 3.**
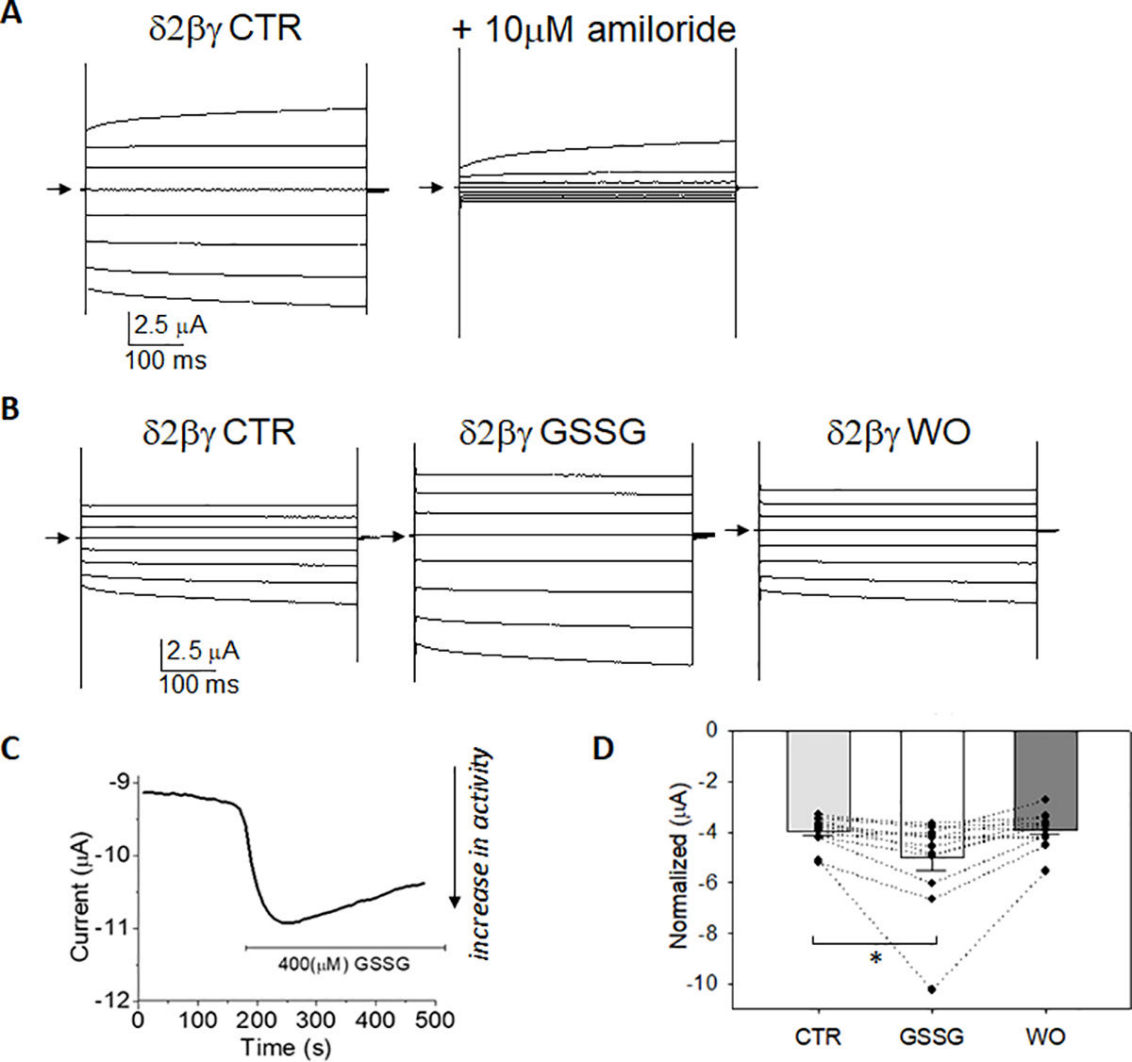
Glutathione disulfide (400 μM) increases δ2βγ-ENaC whole cell current **A)** Representative amiloride-sensitive δ2βγ-ENaC whole cell current; arrow indicates 0 current. Test pulses between −80 to +60 mV shown in 20 mV increments. **B)** GSSG perfusion increases δ2βγ-ENaC current and returns to basal levels following washout (WO). **C)** Representative time course of GSSG induced activation of δ2βγ-ENaC whole cell current plotted at −60 mV holding potential. **D)** Normalized whole cell current of CTR and GSSG treated δ1βγ-ENaC expressing oocytes at −80 mV, wherein GSSG increased current from δ2βγ-ENaC expressing oocytes from −3.9 ± 0.16 μA to −5.0 ± 0.50 μA; n=13 independent observations from 3 different oocyte preparations, *=*p*<0.05 using Paired t-test comparisons.

In Figures 2 and 3, we examined the response of δ1βγ-ENaC and δ2βγ-ENaC whole cell current in the presence of 400 μM GSSG, and following washout. Representative voltage-clamp studies between −80 mV and +60 mV in 20 mV step intervals are shown for δ1βγ- and δ2βγ-ENaC expressing oocytes (Figures 2B and 3B, respectively). These figures show that extra-cellular perfusion of GSSG increases whole cell current when the δ-subunit replaces the α-subunit. Figures 2C and 3C are representative traces showing that δβγ channels respond to GSSG with a transient increase in whole cell current within 10 seconds of perfusion. Normalized whole cell current in Figures 2D and 3D indicate that δ1 or δ2 subunit replacement of the α-ENaC subunit leads to increases in whole cell current following GSSG perfusion; these effects are opposite the GSSG-mediated decrease in αβγ-ENaC whole cell current shown in Figure 1.

### Small airway epithelial cells express α, β, γ and δ-ENaC subunits with varied phenotypic responses to GSSG

**Figure 4.**
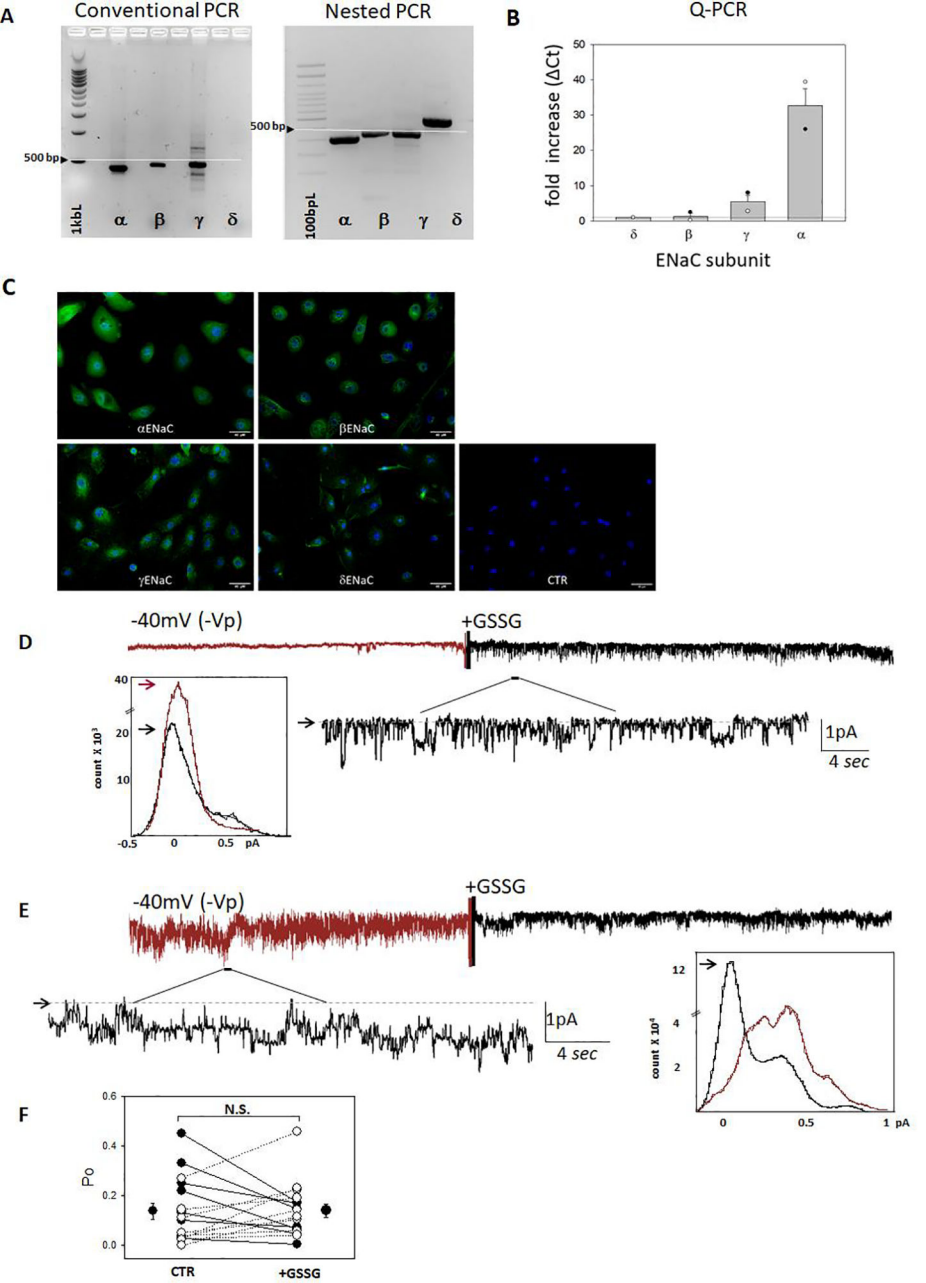
Human small airway epithelial cells express α, β, γ and δ-ENaC subunits **A)** Conventional and nested PCR analysis of α-, β, γ- and δ-ENaC transcript expression in hSAEC on 1.2% agarose gel; arrow indicates 500 base pair (bp) molecular size indicator. **B)** Quantitative real time PCR determination of threshold cycles (ΔCt) for α-, β-, γ- and δ-ENaC transcript expression in hSAEC. **C)** Immuno-histochemical detection of α-, β-, γ- and δ-ENaC protein in hSAECs as indicated. Nuclei were labeled with DAPI. No primary antibody was added to the control (CTR) panel to show that positive signals are not due to non-specific binding of the secondary goat anti-rabbit IgG-Alexa Fluor 488 antibody. Fluorescence signal threshold defined using CTR settings and remained consistent for all subunits analyzed. **D,E)** Representative cell-attached single channel analysis of hSAEC before (red trace) and following 400 μM GSSG application to extracellular bath. A portion of GSSG-induced increase in activity is enlarged to show single channel detail; downward deflections from arrow indicates channel opening. Histogram in the inlay shows freguency of channels in closed (0 pA) or open state. GSSG increased activity as shown in panel D, but decreased activity in panel E. **F)** Distinct and varied response of hSAECs to GSSG; 44% of single channel recordings showed a GSSG-induced decrease in ENaC Po, and 56% of the recordings showed a GSSG-induced increase in ENaC Po; n=16 independent patch-clamp recordings from hSAECs.

**Figure 5.**
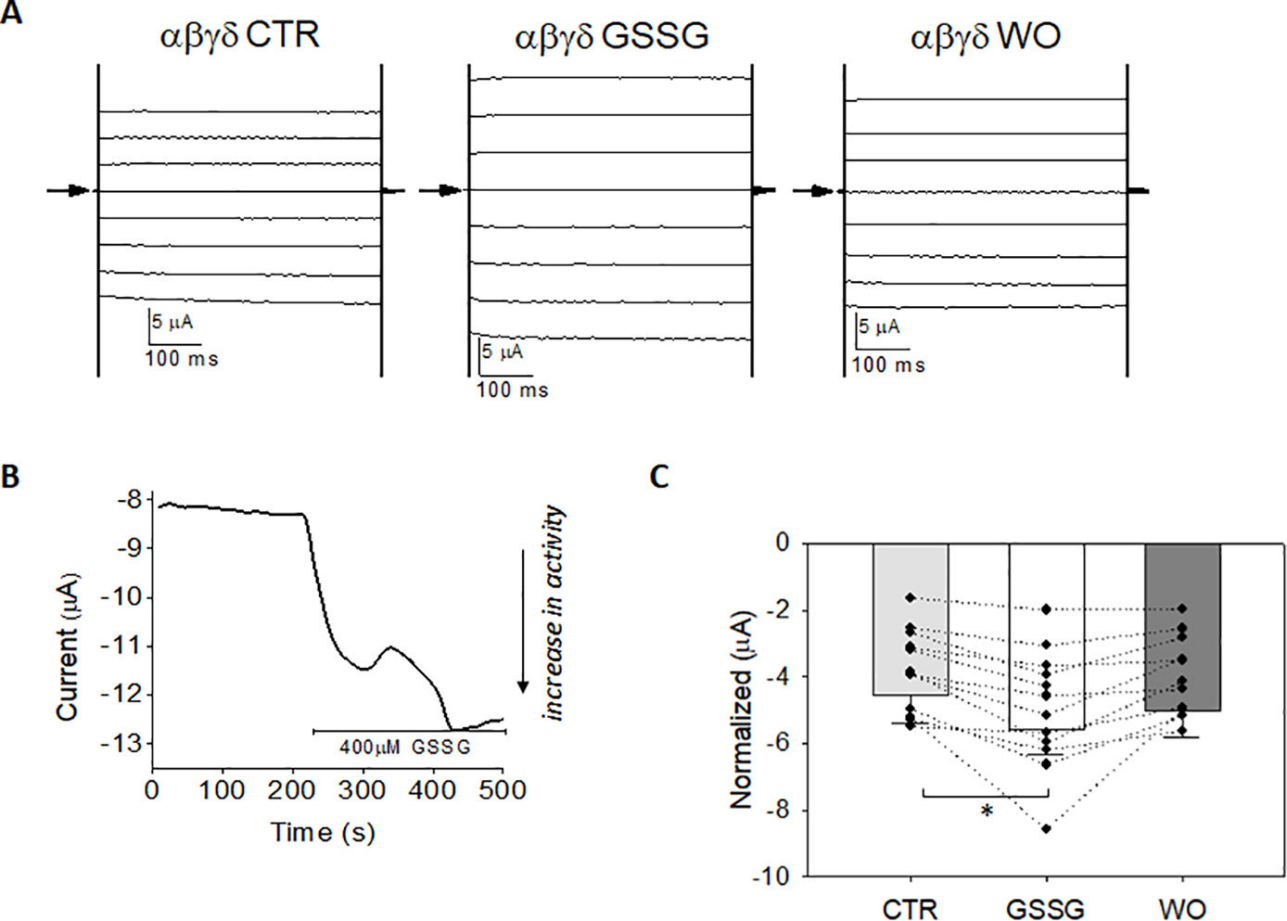
Glutathione disulfide (400 μM) increases αβγδ-ENaC whole cell current **A)** GSSG perfusion increases αβγδ-ENaC cell current, which returns to baseline levels following wash out (WO); arrow indicates 0 current. Voltage steps between −80 to +60 mV shown in 20 mV increments. **B)** Representative time course of GSSG induced activation of αβγδ-ENaC whole cell current plotted at 60 mV holding potential. **C)** Normalized current of CTR and GSSG treated aPyb-ENaC expressing oocytes at −80 mV, wherein GSSG decreased current from −4.5 ± 0.80 pA to −5.58 ± 0.7 μA; n=13 independent observations from 2 different oocyte preparations, *=*p*<0.05 using Paired t-test comparisons. GSSG washout returned whole cell current to baseline levels in each observation.

In cells that express α, β, γ and δ-ENaC subunits [7,20], it is difficult to reconcile what trimeric structure would form at the apical membrane [21,22], and whether subunit composition could impact fluid homeostasis. In Figure 4, we show that hSAECs express α, β, γ and δ-ENaC subunits using conventional, nested, and quantitative PCR (Figures 4A, 4B). Immunohistochemistry labelling of α, β, γ and δ-ENaC protein in fixed hSAECs confirms PCR findings (Figure 4C). Single channel patch clamp analysis of hSAECs indicate that the cellular response to GSSG varies distinctively (Figures 4D–4F). Figure 4D shows a representative recording wherein GSSG increases ENaC activity; the histogram (inset) shows an increase in a 0.6 pA channel with a calculated conductance of 14pS following GSSG application to the extracellular bath. However, in a separate cell attached hSAEC recording (Figure 4E), GSSG decreases ENaC activity (as shown in the cell attached recording and histogram). Figure 4F shows that because of the varied and opposing cellular responses to GSSG measured at the single channel level, there would be no expected change in net sodium transport across the ltmg epithelia; 7 recordings showed a decrease in activity and 9 recordings showed an increase in activity. Although the reason for these results are unclear, we postulated that the observed effects could be attributed to hSAEC’s expression of four ENaC subunits. In order to model α, β, γ and δ expression in hSAECs, we co-expressed all four ENaC cRNAs (in a 1:1:1:1 ratio) in oocytes. Figure 5 shows that this led to assembly of a sodium channel that responds to GSSG with an increase in whole cell current, suggesting that δβγ-channels have assembled at the surface membrane to influence net sodium uptake.

### GSSG post-translational modification of δ1- and δ2-ENaC

**Figure 6.**
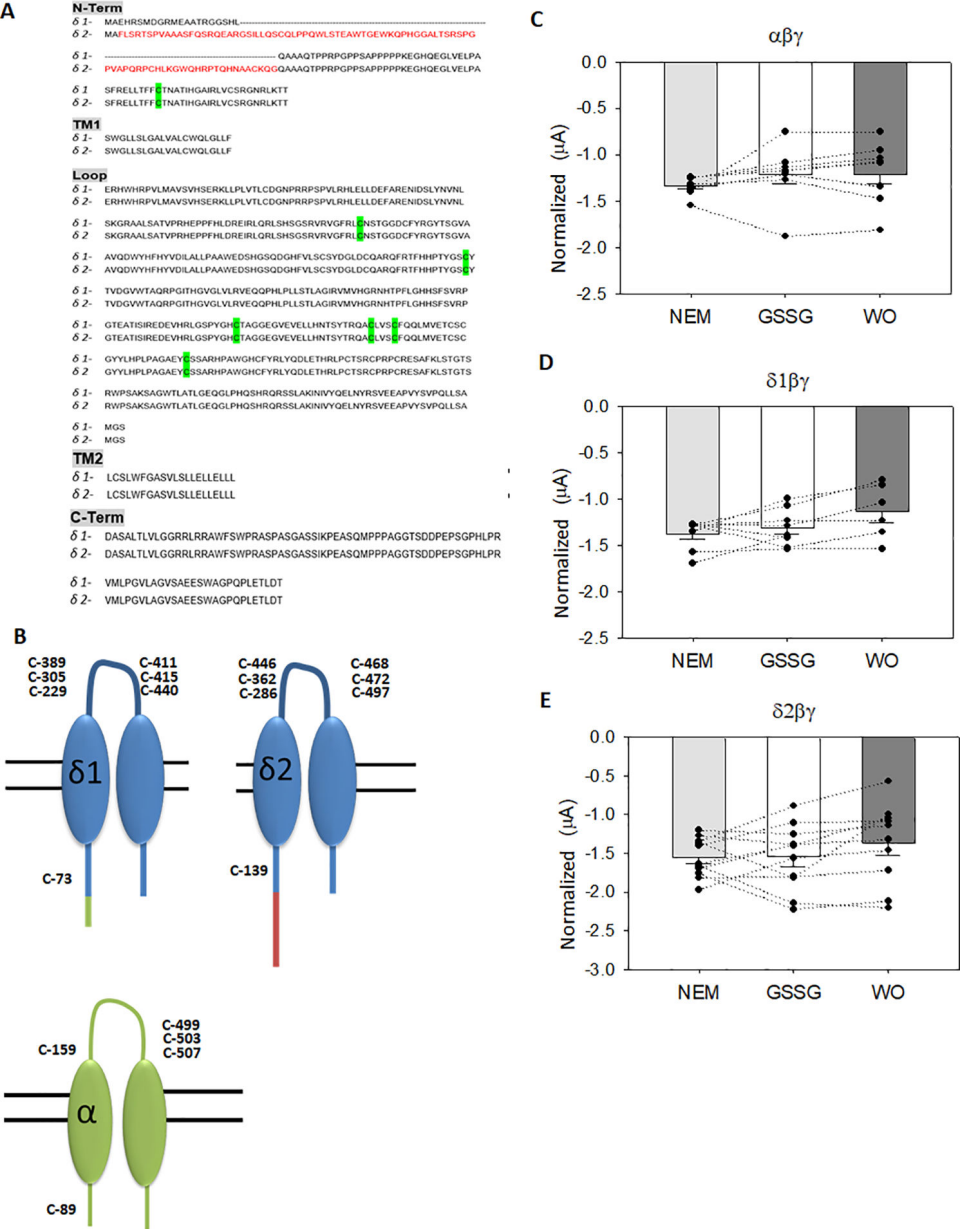
*In silico* analysis of δ-ENaC S-glutathionylation **A)** Amino acid sequence of N-terminal, transmembrane domains 1 and 2, extracellular loop, and C-terminal domains of δ1 and δ2 ENaC subunits with predicted protein-SSG site highlighted. **B)** Illustration showing site of predicted Cys glutathionylation in α, δ1, and δ2 ENaC isoforms. C-E) 10 μM NEM pretreatment attenuates GSSG-mediated changes in ENaC whole cell current, αβγ, N=11, δ1βγ, N=11, δ2βγ N=8 from 3 different batches of oocytes. All whole cell currents were normalized to current amplitudes at −60 mV prior to NEM treatment; *=*p*<0.05 using Paired *t*-test comparisons.

In Figures 6A, 6B, we identified δ1- and δ2 Cys thiols which may be post-translationally modified using *in silico* analysis. Figures 6C–6E shows that N-Ethylmaleimide (NEM) covalent modification of all Cys residues in oocytes expressing αβγ, δ1βγ, and δ2βγ ENaC attenuated GSSG induced effects previously observed in Figures 1–3. Together, the outcomes from our studies show that Cys thiol modification may play an important role in regulating δβγ and αβγ channels, albeit in an opposing manner.

## Discussion

### GSSG regulates αβγ and αβγ-ENaC

Many functional studies indicate that δ- subunit replacement of α-ENaC can lead to divergent biophysiological responses. Capsazepine, a synthetic compound commonly used as a competitive antagonist for transient receptor potential vanilloid subfamily 1 (TRPV1) increases δ1βγ and δ2βγ ENaC activity, but inhibits αβγ channel [7,23]. Evans blue has been described as a specific antagonist of δβγ-ENaC in heterologous expression systems [24]. On the contrary, Evans blue has been noted for its transient increase in human αβγ ENaC activity [7]. Moreover, extracellular protons activated δ-ENaC expressing channels [25,26], but had no effect on αβγ-ENaC activity [27]. Herein, we found that GSSG increases both δ1βγ- and δ2βγ-ENaC activity (Figure 2,3) but inhibits αβγ-channels (Figure 1). Our findings are in line with the aforementioned studies reporting various differences in the sensitivities of αβγ- and δβγ-ENaCs to both synthetic compounds and endogenous signalling regulators [7,23–27]. In our studies, we found that GSSG increased or decreased ENaC whole cell current by approximately 27% depending on whether δ- or α- subunit, respectively, were co-expressed with βγ-ENaC in oocytes. Given the miniscule amount of fluid lining the lung surface area, even small changes in lung ENaC activity can lead to substantial change in extracellular fluid volumes. Presently, it is difficult to predict the direction of vectorial Na^+^ transport in vivo following GSSG exposure based on our single channel analysis which revealed that ENaC responds to GSSG non-uniformly. It is interesting, however, to note that αβγδ-ENaC expressing oocytes showed an increase in current (an effect similar to that measured in δβγ-ENaC expressing oocytes). Also, intriguing is that quantitative PCR showed that hSAECs express less δ-subunits than α-subunits, yet 56% of the single channels recordings responded to GSSG with increase in ENaC activity, albeit, there was no overall significant change in ENaC activity. The signalling mechanisms responsible for controlling δβγ- and αβγ-ENaC’s differential response to GSSG remains unknown and is the focus of future research efforts in our laboratory.

### GSSG and post-translational modification of ion channels

S-glutathionylation is the reversible formation of mixed disulfides between GSSG and cysteine residues in proteins. Support from the literature shows that S-glutathionylation can occur within a single cysteine residue or involve multiple residues. Kir 6.1 is post-translationally regulated at Cys 176 and 43 in the membrane spanning helix domain; and Kir 5.1 is S-glutathionylated at Cys 158, located within the inner helix of the channel [28]. Cys 1344 in the second nucleotide binding domain of the cystic fibrosis transmembrane conductance regulator (CFTR) has been identified as the primary site for S-glutathionylation [29]. The KATP β-1 subunit has also been shown to be S-glutathionylated at Cys 46 [30]. Together, these studies indicate that S-glutathionylation is an important post-translational modifier of ion channels. Each of the ENaC subunits express several conserved Cys residues, however, the precise site of redox sensitivity has not been characterized. Herein, *in silico* analysis of δ1- and δ2-ENaC predicted putative S-glutathionylated sites in δ-ENaC isoforms. Figure 6A illustrates the 6 cysteine residues in the extra-cellular loop, and a single cysteine residue in the amino-terminal domain of the δ1 and δ2 ENaC subunits that may be GSSG sensitive (Figure 6B). In comparison, the α-ENaC subunit has a similar number of predicted residues that may be S-glutathionylated. The quick (within seconds) change in αβγ and δβγ ENaC whole cell current suggests that the extracellular Cys residues may be the targeted site of GSSG modification. However, the differential effects elicited by GSSG between the two channel types (αβγ vs δβγ) indicates that the Cys residues in the less conserved amino terminal region could also serve as the key regulatory site. Systematic evaluation of δ- and α- Cys residues and their role in sodium channel regulation is the focus of future research efforts in our lab.

### Physiological importance of GSSG signalling and ENaC in the lung

Ion channels and transporters play critically important roles in human diseases, such as cystic fibrosis (CF). It is clear that the loss of CFTR function and hyperactive ENaC activity results in airway surface liquid dehydration contributing to airway obstruction and bacterial colonization in CF [31–33]. Although CFTR has been shown to be S-glutathionylated [29], it is unclear whether ENaC is similarly regulated, and whether the altered GSH/GSSG redox potential of CF airway epithelial cells [34,35] play important roles in the pathogenesis of the disease by altering ion channel and transporter function. Based on experimental outcomes from this study, we surmise that under the pathological condition of CF, it may be advantageous to shift subunit composition of lung epithelial sodium channels from δβγ-ENaC to αβγ-ENaC given that GSSG attenuates net Na^+^ absorption in αβγ-channels. This selective expression of subunit expression could mitigate the excessively thick mucus plugs in CF lung. While this idea represents speculative discussion, there has indeed been association between milder lung disease phenotype in CF patients expressing a δ1ENaC subunit mutation that has been reported in the literature [36].
